# Salivary Cytokine Profiles in Active Smokers: An Exploratory Cross-Sectional Study of IL-2, IL-6, and IL-8

**DOI:** 10.3390/diagnostics16142232

**Published:** 2026-07-16

**Authors:** Efstathios Grammatikis, Liliana Sachelarie, Corina Laura Ștefănescu, Marius-Daniel Radu, Cristina Furnică, Raluca Ozana Chistol, Elena Șapte

**Affiliations:** 1 Department of Anatomy, Faculty of Dentistry, Ovidius University of Constanța, 900527 Constanța, Romania; stathisgrammatikis@icloud.com (E.G.); esapte@univ-ovidius.ro (E.Ș.); 2 Department of Dental Medicine, Apollonia University, 700511 Iași, Romania; 3 Faculty of Natural and Agricultural Sciences, Ovidius University of Constanța, University Alley No. 1, Campus, Building B, 900470 Constanța, Romania; marius.radu@univ-ovidius.ro; 4 Discipline of Anatomy, Faculty of Medicine, Grigore T. Popa University of Medicine and Pharmacy, University Street 16, 700115 Iași, Romania; cristina.furnica@umfiasi.ro (C.F.); raluca-ozana.chistol@umfiasi.ro (R.O.C.)

**Keywords:** salivary biomarkers, oral inflammation, smoking, interleukin-2, interleukin-6, interleukin-8, cytokine profiling, immune regulation, oral health diagnostics, saliva

## Abstract

**Background:** Active smoking is recognized as a major risk factor for oral and systemic inflammatory disorders. Tobacco smoke can alter immune responses and modulate the production of pro-inflammatory cytokines, potentially contributing to periodontal tissue damage and impaired oral health. Salivary biomarkers represent a non-invasive and clinically accessible approach for monitoring inflammatory processes. The present study aimed to evaluate salivary levels of interleukin-2 (IL-2), interleukin-6 (IL-6), and interleukin-8 (IL-8) in active smokers and non-smokers and to explore their potential relevance as indicators of oral inflammatory status. **Methods:** A total of 98 clinically healthy adults aged 20–72 years were included in this cross-sectional study. Participants were divided into four groups according to smoking status and sex: female controls, male controls, female smokers, and male smokers. Unstimulated saliva samples were collected using standardized procedures and analyzed for IL-2, IL-6, and IL-8 concentrations using commercially available ELISA kits. Statistical analyses were performed using SPSS, with significance set at *p* < 0.05. **Results:** Salivary cytokine profiles differed between smokers and non-smokers across all investigated biomarkers. Female smokers exhibited lower mean IL-8 levels than female controls, whereas male smokers showed slightly lower IL-8 concentrations than male controls. IL-6 concentrations tended to be higher in female smokers and lower in male smokers relative to their respective control groups. Similarly, IL-2 levels displayed modest variations between smokers and non-smokers. However, none of the observed differences were statistically significant (*p* > 0.05). A significant positive correlation was identified between salivary IL-2 and IL-6 concentrations (r = 0.559, *p* < 0.001). **Conclusions:** Although statistically significant differences were not identified, the observed trends suggest that active smoking may influence salivary cytokine profiles associated with oral inflammatory responses. Salivary IL-2, IL-6, and IL-8 remain promising biomarkers for the non-invasive assessment of inflammatory status and warrant further investigation in larger cohorts. Future studies incorporating additional clinical and periodontal parameters are needed to clarify the relationship between smoking-related immune modulation and oral inflammatory processes.

## 1. Introduction

Inflammation represents one of the most important biological defense mechanisms involved in maintaining tissue homeostasis and protecting the host against external and internal harmful stimuli. Cytokines play a central role in regulating inflammatory and immune responses, coordinating communication between immune cells and modulating the intensity and duration of inflammatory processes. Among these cytokines, interleukin-2 (IL-2) is recognized as a key regulator of T-cell proliferation, differentiation, and immune homeostasis [1].

Smoking remains one of the most important preventable causes of morbidity and mortality worldwide. In addition to its well-established association with cardiovascular, respiratory, and neoplastic diseases, tobacco consumption has profound effects on immune function and inflammatory regulation. Recent proteomic investigations have demonstrated that smoking induces significant alterations in salivary proteins and inflammatory mediators, suggesting a direct impact on oral immune homeostasis [2].

In recent years, saliva has attracted considerable attention as a diagnostic biological fluid due to its non-invasive collection, ease of sampling, and ability to reflect both local and systemic physiological changes. Several studies have demonstrated the utility of salivary biomarkers in monitoring oral, infectious, inflammatory, and systemic diseases [3]. Furthermore, salivary cytokine analysis has been proposed as a valuable approach for identifying pathological alterations before the appearance of clinically detectable signs and symptoms [4].

The oral cavity represents a complex biological environment constantly exposed to microbial, chemical, and mechanical challenges. Under physiological conditions, immune homeostasis is maintained through a balanced interaction between host defense mechanisms and resident microbiota. However, chronic exposure to tobacco smoke may disrupt this equilibrium and contribute to the development of inflammatory alterations within periodontal and oral tissues [5].

Interleukin-8 (IL-8) is one of the most important chemokines involved in neutrophil recruitment and activation during inflammatory responses. Elevated IL-8 levels have been associated with periodontal tissue destruction and chronic inflammatory conditions affecting the oral cavity [6]. Similarly, interleukin-6 (IL-6) is a multifunctional cytokine involved in inflammatory signaling, immune regulation, and bone metabolism [7]. Although IL-8 and IL-6 have been extensively investigated in inflammatory diseases, their response to chronic tobacco exposure remains incompletely understood [8].

Recent evidence suggests that cytokine-based biomarkers may contribute to the diagnosis and monitoring of oral inflammatory conditions. Systematic reviews have demonstrated that salivary biomarkers may reflect pathological processes occurring in periodontal tissues and periapical lesions, supporting their potential diagnostic value [9]. Several studies have specifically identified IL-8 as a biomarker associated with periodontal disease severity and inflammatory burden [10].

Tobacco smoke contains numerous toxic compounds capable of altering cytokine production and immune cell function. Experimental investigations have shown that cigarette smoke extracts can suppress the production of several cytokines, including IL-2, through direct effects on immune cells [11]. In parallel, IL-6 has emerged as a critical mediator linking local inflammation to tissue destruction and systemic complications associated with periodontal disease [12].

The growing interest in salivary diagnostics has stimulated research to identify reliable biomarkers that reflect oral inflammatory status. Salivary biomarker panels have been proposed as promising tools for monitoring oral pathologies and assessing host inflammatory responses [13]. In addition, advances in immunological research have significantly expanded our understanding of IL-2 biology and its role in immune regulation [14,15].

Several studies have demonstrated that cigarette smoke may impair immune function by suppressing cytokine production and modulating T-cell responses [16]. Moreover, smoking has been associated with increased periodontal inflammation and altered cytokine profiles within gingival and salivary environments, suggesting a potential contribution to oral tissue damage and disease progression [17].

Despite increasing evidence regarding the effects of smoking on immune regulation, the combined evaluation of salivary IL-2, IL-6, and IL-8 levels in clinically healthy smokers remains insufficiently investigated. Furthermore, the potential relationships among these cytokines, particularly between immune-regulatory and pro-inflammatory mediators, have received limited attention in salivary biomarker research.

Although IL-2, IL-6, and IL-8 have been individually investigated in relation to smoking and oral inflammation [6,11,16,17], the available evidence remains heterogeneous, with most studies focusing on individual cytokines, different biological samples, or patients with established periodontal or systemic diseases [3,6,9,13,17]. Unlike most previous reports, the present study explored the combined salivary profile of IL-2, IL-6, and IL-8 in clinically healthy smokers and included an exploratory sex-stratified analysis. Given the increasing interest in saliva as a non-invasive diagnostic fluid for monitoring oral immune responses [3,13], this approach may contribute to a more comprehensive characterization of smoking-associated inflammatory alterations within the oral environment. Therefore, the primary aim of this exploratory cross-sectional study was to evaluate salivary concentrations of IL-2, IL-6, and IL-8 in active smokers and non-smokers. A secondary exploratory objective was to examine whether these cytokine patterns differed according to sex.

Based on previous evidence showing that tobacco exposure can modulate immune responses and cytokine production, we hypothesized that active smokers would present altered salivary cytokine profiles compared with non-smokers. Specifically, we expected smoking to be associated with changes in IL-2, reflecting immune-regulatory activity, IL-6, reflecting pro-inflammatory signaling, and IL-8, reflecting neutrophil-related inflammatory responses. Given the exploratory nature of the study, we also examined whether these cytokine patterns differed by sex.

## 2. Materials and Methods

### 2.1. Study Design and Participants

This cross-sectional observational study was conducted to evaluate the influence of active smoking on salivary concentrations of interleukin-2 (IL-2), interleukin-6 (IL-6), and interleukin-8 (IL-8). A total of 98 clinically healthy adults aged between 20 and 72 years were included in the investigation.

The study protocol was reviewed and approved by the Institutional Ethics Committee of Ovidius University (Approval No. 622/13 June 2025). All participants were informed of the study’s objectives and procedures and provided written informed consent prior to enrollment. The study was conducted in accordance with the ethical principles of the Declaration of Helsinki and its subsequent amendments concerning research involving human subjects.

Participants were allocated into four study groups according to sex and smoking status:

✓ Female non-smokers (Control Female Group, CTRLF; *n* = 32);
✓ Male non-smokers (Control Male Group, CTRLM; *n* = 15);
✓ Female smokers (Experimental Female Group, ExpF; *n* = 14);
✓ Male smokers (Experimental Male Group, ExpM; *n* = 37).

All participants underwent a clinical examination and completed a structured questionnaire regarding medical history, smoking habits, and general lifestyle characteristics.

### 2.2. Inclusion and Exclusion Criteria

The inclusion criteria consisted of the following:

✓ Age between 20 and 72 years;
✓ Clinically healthy oral status, as determined by routine clinical oral examination performed by experienced dental clinicians, showing no evidence of acute oral infection, active oral mucosal lesions, or clinically apparent periodontal disease;
✓ Willingness to participate in the study and provide informed consent.

The exclusion criteria included the following:

✓ Systemic inflammatory or autoimmune diseases;
✓ Current antibiotic or anti-inflammatory treatment;
✓ Pregnancy or lactation;
✓ Recent oral surgical procedures;
✓ Presence of active oral infections or severe periodontal disease.

### 2.3. Saliva Collection Protocol

Unstimulated whole saliva samples were collected using the passive drooling technique with Salivette^®^ collection devices (Sarstedt, Nümbrecht, Germany). Participants were instructed to refrain from eating, drinking, smoking, or performing oral hygiene procedures for at least two hours before sample collection.

All saliva samples were collected during the morning hours under standardized conditions. Approximately 1.5 mL of fasting saliva was obtained from each participant.

Immediately after collection, samples were stored at 4 °C and subsequently centrifuged at 3000 rpm for 10 min to remove cellular debris and impurities. The resulting supernatants were aliquoted and prepared for biomarker analysis.

### 2.4. Determination of Salivary Cytokines

Salivary concentrations of IL-2, IL-6, and IL-8 were quantified using commercially available enzyme-linked immunosorbent assay (ELISA) kits (FineTest^®^, Wuhan Fine Biotech Co., Wuhan, China) according to the manufacturer’s instructions.

The following assay kits were used:

✓ IL-2 ELISA Kit (Catalog No. EH0189);
✓ IL-6 ELISA Kit (Catalog No. EH0201);
✓ IL-8 ELISA Kit (Catalog No. EH0205).

Absorbance values were measured using a microplate reader, and cytokine concentrations were calculated from standard calibration curves generated for each assay. Final results were expressed as pg/mL.

All assays were performed within the analytical range recommended by the manufacturer. Standard curves and quality control samples were included in each assay run to ensure reliability of the measurements. Samples with values outside the detection range were reanalyzed when necessary. Analytical sensitivity, limits of detection, and intra-assay and inter-assay variability were evaluated in accordance with the manufacturer’s technical specifications.

### 2.5. Statistical Analysis

Statistical analysis was performed using IBM SPSS Statistics version 29.0 (IBM Corp., Armonk, NY, USA). Descriptive statistics were calculated for all variables and are presented as mean ± standard deviation (SD). The normality of data distribution was assessed using the Shapiro–Wilk test, and homogeneity of variances was evaluated using Levene’s test. Comparisons among the four independent study groups were performed using one-way analysis of variance (ANOVA). Statistical significance was established at *p* < 0.05. A post hoc statistical power analysis was performed based on the observed sample size and effect sizes to support the interpretation of the non-significant findings.

## 3. Results

### 3.1. Baseline Characteristics of the Study Population

A total of 98 participants were included in the study, comprising 45 female and 53 male subjects, aged 20 to 72 years. Participants were allocated into four groups according to smoking status and sex: female non-smokers (CTRLF), male non-smokers (CTRLM), female smokers (ExpF), and male smokers (ExpM) (Table 1). The control groups consisted of clinically healthy non-smokers, whereas the experimental groups included clinically healthy active smokers. No participant presented acute oral infections, active mucosal lesions, or severe periodontal disease at the time of examination.

Assessment of statistical assumptions demonstrated that homogeneity of variances was satisfied for all investigated cytokines according to Levene’s test (all *p* > 0.05).

Age differed significantly among the four study groups (one-way ANOVA, *p* = 0.003). Female smokers were older on average than the other study groups, particularly compared with female non-smokers. This age imbalance was considered a potential confounding factor in interpreting the salivary cytokine comparisons.

### 3.2. Comparison of Salivary Cytokine Levels Between Smokers and Non-Smokers

#### 3.2.1. Salivary IL-2 Levels

Salivary IL-2 concentrations were evaluated in smokers and non-smokers according to sex. Female smokers exhibited a mean IL-2 concentration of 9.69 ± 2.55 pg/mL compared with 7.86 ± 1.22 pg/mL in female non-smokers. Similarly, male smokers had a mean IL-2 concentration of 17.55 ± 7.21 pg/mL, whereas male non-smokers had 15.69 ± 9.07 pg/mL (Table 2).

Although slightly higher IL-2 concentrations were observed in smokers, the differences between smoker and non-smoker groups did not reach statistical significance (female groups: *p* = 0.470; male groups: *p* = 0.870).

The results indicate that active smoking was associated with modest variations in salivary IL-2 levels; however, these changes were not statistically significant within the investigated population. One-way ANOVA confirmed the absence of statistically significant differences among the four study groups (*p* = 0.515).

#### 3.2.2. Salivary IL-6 Levels

Salivary IL-6 concentrations were measured in all study groups. Female smokers had slightly higher IL-6 levels (6.17 ± 1.04 pg/mL) than female non-smokers (5.32 ± 0.68 pg/mL). In contrast, male smokers exhibited lower IL-6 concentrations (4.83 ± 0.44 pg/mL) than male non-smokers (9.91 ± 4.97 pg/mL).

Despite these variations, no statistically significant differences were identified between smokers and non-smokers (female groups: *p* = 0.420; male groups: *p* = 0.331) (Table 3).

These findings suggest that smoking status was associated with variability in IL-6 concentrations; however, the observed differences were not statistically significant. Similarly, one-way ANOVA did not identify significant differences among the four study groups (*p* = 0.630).

#### 3.2.3. Salivary IL-8 Levels

Salivary IL-8 concentrations were also analyzed in smokers and non-smokers. Female smokers showed a mean IL-8 concentration of 37.23 ± 5.04 pg/mL compared with 40.58 ± 4.53 pg/mL in female non-smokers. Similarly, male smokers exhibited a mean IL-8 concentration of 44.60 ± 7.52 pg/mL, whereas male non-smokers exhibited 48.64 ± 7.52 pg/mL.

No statistically significant differences were observed between smokers and non-smokers for IL-8 concentrations (female groups: *p* = 0.611; male groups: *p* = 0.729) (Table 4). One-way ANOVA likewise confirmed the absence of statistically significant differences among the four study groups (*p* = 0.301).

Overall, active smoking was associated with a slight reduction in salivary IL-8 levels; however, the observed differences remained statistically non-significant.

The mean differences and corresponding 95% confidence intervals are presented in Table 5. All confidence intervals included zero, supporting the absence of statistically significant differences between smokers and non-smokers for the investigated cytokines.

### 3.3. Correlation Analysis Between Salivary Cytokines

To further investigate the relationships among the evaluated inflammatory biomarkers, Pearson correlation analyses were performed between salivary IL-2, IL-6, and IL-8 concentrations.

A statistically significant, moderate positive correlation was observed between IL-2 and IL-6 levels (r = 0.559, *p* < 0.001), indicating that participants with higher IL-2 concentrations tended to have higher IL-6 concentrations. This association represented the strongest relationship observed among the investigated cytokines and may reflect coordinated activation of immune-regulatory and pro-inflammatory pathways within the oral environment.

No significant correlations were observed between IL-2 and IL-8 (r = 0.109, *p* = 0.313) or between IL-6 and IL-8 (r = 0.184, *p* = 0.086).

Overall, the findings suggest that IL-2 and IL-6 may participate in interconnected inflammatory signaling mechanisms, whereas IL-8 appears to follow a more independent expression pattern in the investigated population (Table 6).

### 3.4. Integrated Assessment of Salivary Cytokine Profiles

To obtain a broader overview of the salivary inflammatory profile, the results of IL-2, IL-6, and IL-8 were interpreted collectively. Although none of the individual cytokines demonstrated statistically significant differences between smokers and non-smokers, the overall cytokine profile revealed distinct trends among the study groups.

Smokers showed small descriptive differences across the biomarkers investigated; however, these findings should be interpreted with caution, as no statistically significant intergroup differences were identified. The most notable finding was the significant positive correlation between IL-2 and IL-6 concentrations, which supports the existence of coordinated inflammatory pathways involving immune regulation and pro-inflammatory signaling mechanisms.

In contrast, IL-8 showed weaker associations with the other cytokines and a tendency toward lower concentrations in smokers, suggesting that its expression may be influenced by additional biological factors independent of the IL-2/IL-6 inflammatory axis.

Taken together, these findings suggest that the simultaneous evaluation of multiple salivary cytokines may provide a more comprehensive characterization of oral inflammatory status than the assessment of individual biomarkers alone. Future investigations incorporating larger study populations, additional inflammatory biomarkers, and detailed periodontal clinical parameters may further improve the understanding of smoking-associated immune alterations within the oral cavity.

## 4. Discussion

The present study evaluated salivary concentrations of IL-2, IL-6, and IL-8 in clinically healthy smokers and non-smokers to investigate the potential influence of tobacco exposure on oral inflammatory biomarkers. Although no statistically significant differences were identified between smokers and non-smokers, several descriptive differences were observed. The observed cytokine patterns, together with the significant correlation between IL-2 and IL-6, may indicate subtle changes in oral immune regulation, even in the absence of clinically evident inflammatory disease.

Salivary cytokines have been increasingly investigated as accessible biomarkers for monitoring oral inflammatory status and host responses to environmental risk factors, including tobacco exposure [18,19]. The modest cytokine variations observed in the present study support the concept that smoking-related immune alterations may occur before the development of overt clinical manifestations.

IL-2 is a central regulator of adaptive immunity, playing a critical role in T-cell activation, proliferation, and immune homeostasis. In the present study, salivary IL-2 concentrations were slightly higher in smokers than in non-smokers in both female and male groups. Although these differences did not reach statistical significance, the observed increases could reflect subtle variations in immune-regulatory pathways, although this interpretation remains exploratory. Previous studies have reported heterogeneous effects of smoking on IL-2 production and immune cell function [20,21]. Experimental investigations have demonstrated that constituents of cigarette smoke, including acrolein and nicotine-derived compounds, may suppress T-cell responses and cytokine production while simultaneously promoting chronic inflammatory activation, resulting in complex and sometimes contradictory immunological patterns [16,22]. Interestingly, smokers exhibited slightly higher mean IL-2 concentrations than non-smokers in both sexes, although these differences were not statistically significant. Because the sex-stratified comparisons were exploratory and based on relatively small subgroup sizes, these findings should be interpreted as hypothesis-generating and require confirmation in larger, balanced cohorts.

Regarding IL-6, female smokers exhibited slightly higher concentrations than female non-smokers, whereas male smokers demonstrated lower values. IL-6 is recognized as a multifunctional cytokine involved in inflammatory signaling, immune regulation, bone metabolism, and tissue remodeling [12,23]. Elevated IL-6 levels have been associated with periodontal disease progression and systemic inflammatory disorders, supporting its role as a key mediator of both local and systemic inflammation [12]. The absence of statistically significant differences in the present study may be explained by the inclusion of clinically healthy participants and by the considerable biological variability commonly reported for salivary IL-6 measurements. Similar findings have been described in previous investigations highlighting the influence of genetic, hormonal, environmental, and behavioral factors on cytokine expression [24,25,26]. Male smokers exhibited lower mean IL-6 concentrations than male controls, although this difference did not reach statistical significance.

The analysis of IL-8 revealed slightly lower concentrations in smokers than in non-smokers across both sexes. IL-8 is an important chemokine responsible for neutrophil recruitment and activation at sites of inflammation and plays a central role in periodontal immune defense [17,26]. Previous investigations have reported altered IL-8 expression in smokers and in patients with periodontal disease, reflecting changes in host defense mechanisms and inflammatory regulation [6,17,27]. However, published findings remain inconsistent, with some studies reporting increased IL-8 concentrations and others showing reduced or unchanged cytokine levels following tobacco exposure [6,17,27,28].

These discrepancies may be explained by methodological differences, including the type of biological sample analyzed, smoking intensity and duration, periodontal status, time since the last cigarette, saliva collection protocol, sample processing, ELISA kit sensitivity, and statistical adjustment for confounding factors. An important aspect to consider is the influence of smoking on the oral microbiological ecosystem. Tobacco exposure has been shown to alter both the composition of the oral microbiome and the inflammatory environment of the oral cavity [29]. Recent studies have demonstrated significant microbial and immunological differences between smokers and non-smokers, suggesting that smoking-associated changes in cytokine expression may occur indirectly through modifications of host–microbe interactions [22,30]. Such mechanisms could contribute to the subtle cytokine variations observed in the present study and may partially explain the variability observed among individual participants.

**Figure 1:**
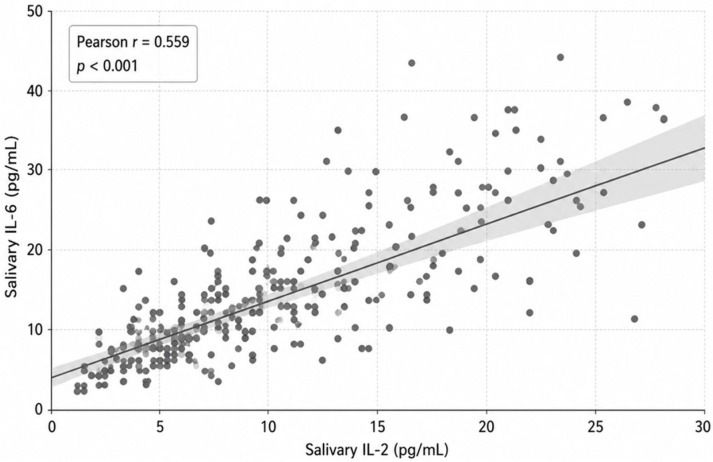
Correlation between salivary IL-2 and IL-6 concentrations.

One of the most noteworthy findings of the present investigation was the significant positive correlation identified between IL-2 and IL-6 concentrations (r = 0.559, *p* < 0.001). As illustrated in Figure 1, participants with higher IL-2 concentrations tended to exhibit higher IL-6 levels, which may reflect coordinated activity of immune-regulatory and pro-inflammatory pathways within the oral environment.

Although the biological significance of this association warrants further investigation, it may support the notion that oral inflammatory responses are governed by interconnected cytokine networks rather than by isolated biomarker alterations. In contrast, no significant correlations were identified between IL-2 and IL-8 or between IL-6 and IL-8, suggesting that IL-8 expression may be influenced by additional regulatory mechanisms independent of the IL-2/IL-6 inflammatory axis.

From an immunological perspective, the positive association between IL-2 and IL-6 may reflect a link between adaptive immune regulation and pro-inflammatory signaling within the oral environment. IL-2 is mainly produced by activated T lymphocytes and plays an essential role in T-cell proliferation, survival, and immune homeostasis. In contrast, IL-6 is produced by several immune and stromal cells and contributes to inflammatory amplification, B-cell differentiation, acute-phase responses, and tissue remodeling. Therefore, the parallel variation in IL-2 and IL-6 observed in the present study suggests that immune-regulatory and inflammatory pathways are not acting independently but are part of a coordinated cytokine response. In clinically healthy smokers, such an association could reflect subtle immune activation occurring before clinically evident oral inflammation. However, because the present study was cross-sectional and did not include cellular immune profiling, periodontal inflammatory indices, or additional cytokines, this interpretation remains hypothesis-generating and should not be considered mechanistic proof.

Several limitations should be acknowledged. First, the sample size was relatively limited and the distribution of participants across the four study groups was unequal, particularly after stratification by sex and smoking status. These aspects may have reduced the statistical power of intergroup comparisons and may limit the generalizability of subgroup-specific findings. In line with this, post hoc power analysis demonstrated limited statistical power (1 − β ranging from 0.05 to 0.21), reflecting the small observed effect sizes. Therefore, the absence of statistically significant differences should be interpreted with caution and does not necessarily rule out subtle biological effects.

Second, smoking exposure was classified only according to current smoking status, without quantitative assessment of cigarettes smoked per day, smoking duration, cumulative exposure expressed as pack-years, nicotine dependence, or time since the last cigarette. These variables may influence salivary cytokine levels and should be incorporated into future studies.

Third, detailed periodontal clinical parameters, including plaque index, gingival index, bleeding on probing, probing depth, and clinical attachment level, were not systematically recorded. Therefore, subtle subclinical periodontal variations among participants cannot be entirely ruled out.

Finally, only three cytokines were evaluated, whereas oral inflammatory responses involve complex interactions among numerous immune mediators and signaling pathways. The cross-sectional design also precludes establishing causal relationships between smoking and cytokine expression.

In addition, potential confounding factors such as alcohol consumption, body mass index, oral hygiene practices, medication use, mild systemic diseases, and dietary habits were not systematically recorded or controlled and therefore may have influenced salivary cytokine concentrations. Future studies should incorporate these variables to better isolate the specific effects of smoking on oral inflammatory biomarkers.

Given the well-established systemic consequences of smoking and its association with increased morbidity and mortality across multiple disease categories [31,32], the identification of reliable salivary biomarkers may provide valuable opportunities for early risk assessment and oral health monitoring. Future investigations involving larger cohorts, broader biomarker panels, quantitative smoking exposure parameters, comprehensive periodontal assessments, and longitudinal follow-up designs are warranted to further clarify the relationship between tobacco use and oral inflammatory status.

## 5. Conclusions

The present study evaluated salivary concentrations of IL-2, IL-6, and IL-8 in clinically healthy smokers and non-smokers. Although no statistically significant differences were identified between groups, small descriptive differences in cytokine concentrations were observed, suggesting that smoking may subtly influence oral immune regulation.

A significant positive correlation between IL-2 and IL-6 concentrations (r = 0.559, *p* < 0.001) was identified, supporting the existence of coordinated inflammatory pathways within the oral environment.

These findings support the potential utility of saliva as a non-invasive biological fluid for monitoring inflammatory biomarkers and investigating smoking-associated immune responses. Further studies involving larger cohorts, quantitative smoking exposure assessment, and broader biomarker panels are warranted to clarify the relationship between tobacco use and oral inflammatory status.

## Figures and Tables

**Table 1:** Baseline characteristics of the study population.

Characteristic	CTRLF	CTRLM	ExpF	ExpM	Total	*p*-Value
Smoking status	Non-smoker	Non-smoker	Smoker	Smoker	—	—
Sex	Female	Male	Female	Male	—	—
Number of participants, *n*	32	15	14	37	98	—
Age (years), mean ± SD	32.2 ± 11.6	31.1 ± 6.0	46.6 ± 15.6	38.2 ± 14.5	36.2 ± 13.6	0.003
Clinical oral status	Healthy	Healthy	Healthy	Healthy	—	—

**Table 2:** Salivary IL-2 concentrations in smokers and non-smokers.

Group	IL-2 (pg/mL), Mean ± SD	*p*-Value
CTRLF	7.86 ± 1.22	
ExpF	9.69 ± 2.55	0.470
CTRLM	15.69 ± 9.07	
ExpM	17.55 ± 7.21	0.870

**Table 3:** Salivary IL-6 concentrations in smokers and non-smokers.

Group	IL-6 (pg/mL), Mean ± SD	*p*-Value
CTRLF	5.32 ± 0.68	
ExpF	6.17 ± 1.04	0.420
CTRLM	9.91 ± 4.97	
ExpM	4.83 ± 0.44	0.331

**Table 4:** Salivary IL-8 concentrations in smokers and non-smokers.

Group	IL-8 (pg/mL), Mean ± SD	*p*-Value
CTRLF	40.58 ± 4.53	
ExpF	37.23 ± 5.04	0.611
CTRLM	48.64 ± 7.52	
ExpM	44.60 ± 7.52	0.729

**Table 5:** Adjusted 95% confidence intervals and effect sizes.

Biomarker	Comparison	Mean Difference (pg/mL)	Adjusted 95% CI (ANOVA)	Effect Size (Cohen’s d)
IL-2	ExpF vs. CTRLF	1.83	−2.34 to 6.00	0.25 (Small)
IL-2	ExpM vs. CTRLM	1.86	−3.12 to 6.84	0.24 (Small)
IL-6	ExpF vs. CTRLF	0.85	−2.48 to 4.18	0.18 (Negligible)
IL-6	ExpM vs. CTRLM	−5.08	−11.23 to 1.07	0.31 (Small)
IL-8	ExpF vs. CTRLF	−3.35	−11.38 to 4.68	0.15 (Negligible)
IL-8	ExpM vs. CTRLM	−4.04	−11.19 to 3.11	0.54 (Medium)

**Table 6:** Correlation analysis among salivary cytokines.

Variables	Pearson r	*p*-Value
IL-2 vs. IL-6	0.559	<0.001
IL-2 vs. IL-8	0.109	0.313
IL-6 vs. IL-8	0.184	0.086

## Data Availability

The data presented in this study are available from the corresponding author upon reasonable request.

## Abbreviations

The following abbreviations are used in this manuscript:

CTRLF	Control Female Group (female non-smokers)
CTRLM	Control Male Group (male non-smokers)
ExpF	Experimental Female Group (female smokers)
ExpM	Experimental Male Group (male smokers)
